# How new approach methods are reshaping virology research

**DOI:** 10.1128/jvi.01326-25

**Published:** 2026-03-16

**Authors:** Min Liu, Jonathan G. Faris, Amanda R. Panfil, Cody J. Warren

**Affiliations:** 1 Department of Veterinary Biosciences, The Ohio State University 2647, Columbus, Ohio, USA; 2 Department of Chemical and Biological Engineering, University of Colorado Boulder 1877, Boulder, Colorado, USA; 3 Infectious Diseases Institute, The Ohio State University 2647, Columbus, Ohio, USA; Universiteit Gent, Merelbeke, Belgium

**Keywords:** new approach methods (NAMs), model systems, translational virology, host-virus interactions

## Abstract

Animal models are a cornerstone of basic and translational virology research, widely used to study viral pathogenesis and evaluate vaccines and therapeutics. However, growing ethical and scientific concerns, alongside recent National Institutes of Health (NIH) and Food and Drug Administration (FDA) initiatives, are accelerating a shift toward non-animal, human-relevant alternatives. Advanced cell- and tissue-based systems now offer powerful platforms to model human disease. This Gem outlines emerging tools and highlights their promise for virology research in a rapidly evolving regulatory and technological landscape.

## INTRODUCTION

In 2025, the two most influential U.S. research agencies marked a turning point in biomedical research by formally shifting funding priorities away from reliance on animal models and toward human-based research approaches. The National Institutes of Health (NIH) ended funding calls restricted to animal studies and has sought to establish a new Office of Research Innovation, Validation, and Application (ORIVA) to accelerate the development and adoption of human-based alternatives (1). Concurrently, the Food and Drug Administration (FDA) released a roadmap encouraging drug developers to replace animal safety studies with human-relevant technologies and advanced computational models, collectively referred to as “new approach methods” or NAMs (2). As a community of scientists, it is imperative to embrace these emerging technologies wherever possible to reduce reliance on animal research. At the same time, it is important to recognize that whole-organism systems remain essential for understanding complex physiology, immune responses, and disease dynamics that cannot yet be fully captured *in silico* or *in vitro*. This Gem will highlight human-based approaches at the forefront of virology research while also emphasizing the critical roles that animal models continue to play in biomedical science.

## UNDERSTANDING “NEW APPROACH METHODS”

**Fig 1:**
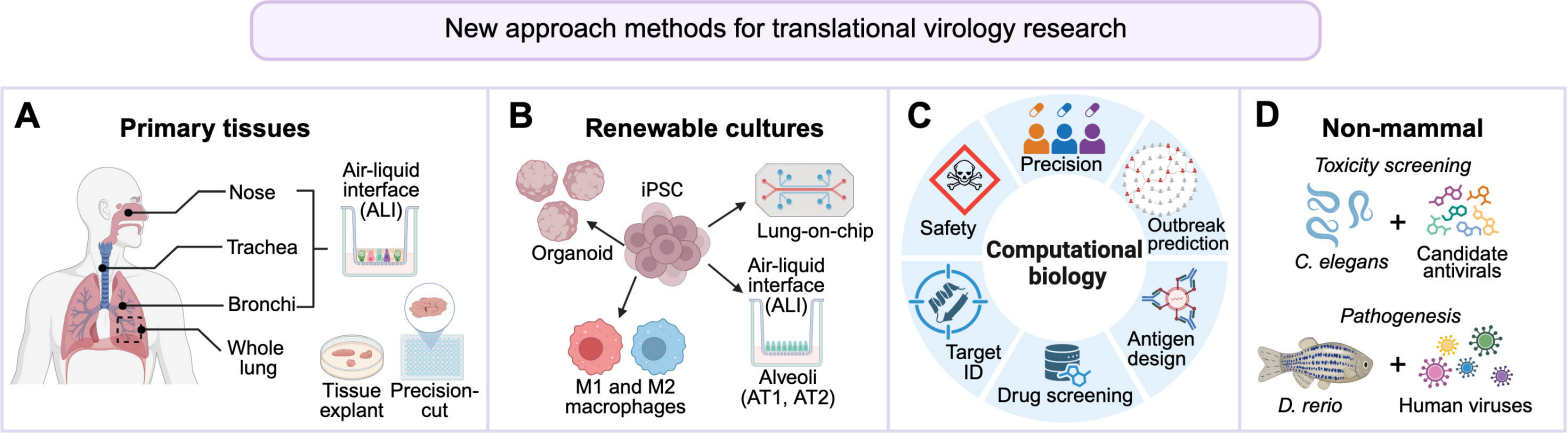
Representative new approach methods (NAMs) used in translational virology research. (**A**) Primary human lung tissues support the development of differentiated air-liquid interface cultures, permitting viral exposure from the apical “airway” surface to better mimic natural infection. Tissue explants and precision-cut lung slices preserve native cellular architecture and resident immune populations, increasing physiological relevance. (**B**) Renewable human cell systems, including induced pluripotent stem cell (iPSC)-derived respiratory epithelium and immune cell subsets, provide scalable platforms when primary tissues are limited. (**C**) Computational NAMs incorporate data-driven modeling tools to predict viral behavior, identify therapeutic candidates, and support vaccine design, as well as forecast transmission and immune escape dynamics. (**D**) Non-mammalian whole-organism models (e.g., *Caenorhabditis elegans*, *Danio rerio*) enable scalable *in vivo* testing of candidate antivirals and host-virus interactions while reducing reliance on traditional mammalian systems. Created in BioRender. Warren, C. (2026) https://BioRender.com/snugfy0.

Despite decades of adherence to the “three R” principles—replacement, reduction, and refinement—animal models remain the cornerstone of preclinical research. Yet, the failure rate for translating promising therapeutics from animal testing to effective human treatments remains exceedingly high (3–6), raising concerns about the predictive value of traditional models and the need for new strategies to improve translational success. In response, a growing emphasis has been placed on developing new approach methods (NAMs) that are designed to model human biology and disease without relying on whole-animal systems. NAMs are not a singular technology, but rather a framework of relevant test systems that have broadly applicable utility in biomedicine (Fig. 1). Below, we provide an in-depth overview of respiratory NAMs, where these platforms are among the most developed, and then broaden our scope to additional systems that exemplify their wider utility in virology research.

### NAMs based on primary cell and tissue cultures

Primary cell- and tissue culture-based NAMs involve the isolation of living cells or tissue fragments directly from organisms and their maintenance under physiologically relevant conditions. In respiratory virus research, air-liquid interface (ALI) cultures are among the most widely adopted human-relevant systems. ALI systems are established by isolating human airway epithelial stem cells from the donor tissue and culturing them in trans-well dishes, where the basal surface is exposed to nutrient-rich media and the apical surface to air—conditions that mimic the architecture and microenvironment of the human respiratory tract (7, 8) (Fig. 1A). Under these conditions, airway cells differentiate into a stratified epithelium comprising ciliated and non-ciliated columnar cells, goblet cells, and basal cells, thereby recreating the morphological and functional complexity of human airway mucosal surface (9–12). Human primary ALI cultures have been widely applied to study the biology of diverse DNA and RNA respiratory viruses, providing both mechanistic (13–17) and translational (18–21) insights. Depending on study needs, epithelial cells from the nasal cavity, trachea, or bronchi can be isolated and differentiated at the ALI to model distinct airway regions (9, 19, 22–26). Importantly, ALI cultures preserve epithelial polarity and enable apical viral inoculation, closely mimicking infection of an intact mucosal surface (27–30).

However, while ALI cultures effectively recapitulate key features of the respiratory epithelium, they lack the multicellular complexity and immune components essential for modeling virus-host interactions within the lung microenvironment. In parallel, donor-derived lung explants (31) and precision-cut lung slices (PCLS) (32, 33) have been used to examine aspects of viral infection that extend beyond epithelial-only systems. Although these models present challenges in standardization and experimental manipulation, advances in automated PCLS preparation have improved experimental consistency relative to tissue explant cultures. Reflecting their complementary utility, PCLS models have seen a marked rise in use in recent years (for comprehensive reviews, see [32–37]).

PCLS are generated by inflating lung tissue with liquid agarose, solidifying the tissue on ice, and sectioning it at a pre-defined thickness. Standardization is achieved through reproducible selection of tissue regions for cutting, maintaining consistency in slice thickness, and uniform punching of slices, ensuring consistency across experiments. Viral infections are performed by incubating slices with viral inocula, followed by washing and longitudinal monitoring of viral replication (36, 37). A recent study by Pechous et al. elegantly demonstrated the power of the PCLS model, showing that severe acute respiratory syndrome coronavirus 2 (SARS-CoV-2) replicates rapidly in the human lung tissue and induces proinflammatory responses characteristic of coronavirus disease 2019 (COVID-19) pathology. Notably, these effects were mitigated by antiviral treatment, underscoring the platform’s value for therapeutic drug screening (38). More broadly, PCLS has proven highly versatile—supporting studies on host species susceptibility to emerging influenza virus strains (39), evaluation of oncolytic virus therapies (40), and identification of host factors that influence infection outcomes (41, 42). Finally, advances in cryopreservation now enable long-term storage and on-demand use of PCLS (43). Looking ahead, building next-generation precision-cut tissue biobanks spanning diverse donors and organ systems would ease transitions away from animal models and accelerate translational virology in human-relevant systems.

While primary cell and tissue cultures offer critical physiological advantages, access to human respiratory tissues remains limited by donor availability. Moreover, when tissues are obtainable, variability in donor clinical background (e.g., smoking history, chronic obstructive pulmonary disease [COPD], and underlying infections) and peri-donation management (e.g., medications) can hinder reproducibility, particularly for large studies. These constraints have driven growing interest in renewable cell culture systems, which provide a consistent and replenishable source of physiologically relevant human-derived cells for experimental use.

### NAMs based on renewable cell cultures

A central component of renewable NAM technologies is the use of induced pluripotent stem cells (iPSCs), which can be indefinitely expanded and directed toward multiple respiratory lineages (Fig. 1B). This approach, combined with the virtually limitless expansion potential of a single iPSC line, provides consistent and renewable access to advanced lung NAMs. Moreover, iPSC-based systems are genetically tractable—allowing modification of host genes prior to differentiation (44–46)—thereby enabling targeted investigation of molecular determinants of viral infection and host susceptibility.

Human respiratory organoids derived from iPSCs represent one such NAM that has been widely adopted across the field (47–49). In 2017, Chen et al. first reported the generation of lung bud organoids from iPSCs, which recapitulated features of branching airway and alveolar structures following xenotransplantation and extended three-dimensional (3D) culture. They further demonstrated that infection of these organoids with respiratory syncytial virus (RSV)—a leading cause of pediatric respiratory illness—produced pathological features resembling those observed in infant lungs (50). Subsequent studies from the same group expanded this system to related viruses in the *Paramyxoviridae* family, including human parainfluenza virus type 3 (HPIV3) and measles virus (MeV) (51), establishing this model as a valuable platform for investigating viral pathogenesis in developing lung tissue. Moreover, organoids derived from primary tissue stem cells have emerged as complementary models, supporting infection by additional clinically relevant respiratory viruses like human rhinovirus C (HRV-C), Middle East respiratory syndrome coronavirus (MERS-CoV), and SARS-CoV-2 (52, 53). Together, these findings underscore the versatility of both iPSC- and primary tissue-derived lung organoid systems for investigating respiratory virus biology.

As with all 3D organoid systems, however, certain practical constraints remain. A key limitation of 3D organoid cultures is their dependence on extracellular matrix (ECM) components that surround and support the organoid structure. While necessary for growth and differentiation, the ECM creates a physical barrier that restricts viral or drug access to internal cell layers, often necessitating microinjection or mechanical disruption to expose the apical surface for inoculation (54, 55). To address some of these limitations, lung-on-a-chip models build upon traditional organoid systems by integrating microfluidics and mechanical forces to more faithfully recapitulate lung physiology (56). These platforms continue to advance, incorporating highly differentiated respiratory epithelial and endothelial cells that reflect key cellular targets of respiratory virus infection (57) and can be further populated with tissue-resident and circulating immune cells (58). We and others have shown that iPSCs enable large-scale production of macrophages (59–62), which can be further differentiated toward a tissue-resident, alveolar macrophage-like phenotype (63, 64). Because iPSCs can generate multiple relevant cell types—for example, macrophages and lung organoids—from a single donor with an identical genetic background, they create powerful opportunities to model complex host-virus interactions without the complications of primary donor tissues. Ultimately, these advances point toward the future potential of personalized organ-on-a-chip systems to guide individualized therapeutic selection; however, current platforms remain limited in their ability to fully reflect individualized physiology, including sex-dependent differences (65, 66).

### NAMs offer a breadth of utility across diverse human organ systems

In addition to advancing respiratory virus research, NAMs are reshaping how we study viral diseases across multiple human organ systems. For example, scalable platforms, such as human iPSC-derived cardiomyocytes, have made previously inaccessible cell types readily available for research (67). Their application to SARS-CoV-2 infection models demonstrates how NAMs can capture human-specific disease mechanisms and support therapeutic testing (68–71). Similarly, central nervous system models—including brain organoids, precision-cut tissue slices, and primary neuroimmune cultures—have enabled controlled evaluation of neurotropism and neurovirulence for both zoonotic encephalitic arboviruses (72–78) and human viruses characterized by long-term persistence (79–84), expanding opportunities for therapeutic testing against these highly consequential groups of pathogens. More broadly, physiologically relevant *in vitro* and *ex vivo* models of the gastrointestinal (85), dermal (86), reproductive (87), hepatic (88), renal (89), and immune compartments (90) are enabling the study of virus-host interactions across diverse tissues. As these platforms continue to mature, they will undoubtedly help bridge long-standing gaps between preclinical research and successful therapeutic development.

### NAMs based on computational biology

Computational approaches now represent an essential pillar of NAMs. In today’s “omics” era, expansive genomic, transcriptomic, and proteomic data sets—combined with queryable protein-structure and medicinal chemical libraries—enable rapid *in silico* screening and hypothesis generation prior to studies involving biological specimens. Furthermore, with the rapid advancement of machine learning and artificial intelligence, these *in silico* tools can be applied to a wide range of virological questions, including but not limited to therapeutic discovery (91–94), epidemiological modeling (94–96), and computational organoid models (97–99) (Fig. 1C).

In the context of emerging viral diseases, computational modeling provides an accelerated path for identifying therapeutic and prophylactic candidates. Molecular docking and molecular dynamics simulations estimate binding affinities and interaction kinetics between viral proteins and potential inhibitors, supporting early drug discovery without the cost or animal use of traditional experimental pipelines (100–102). Predictive toxicology tools allow pre-screening for potential adverse effects on human health, even before biological testing occurs (for detailed reviews, see [103–106]). Molecular modeling has been used to predict SARS-CoV-2 spike-ACE2 binding across species to assess reservoir potential (107) and to forecast immune escape by identifying high-risk mutations relevant for vaccine design (108, 109). Beyond vaccine development (110–113), *in silico* models have been a valuable tool to inform vaccine deployment strategies through epidemiological modeling of disease surveillance and spread (95, 114–116). Multiple transmission models aided in the understanding of testing, quarantining, and social distancing strategies during early phases of the COVID-19 pandemic (95, 115, 117), and informed public health officials as to what populations should be prioritized when vaccine supplies were limited (114). In addition to population-level applications, *in silico* tools are becoming increasingly relevant for mechanistic studies of infection. Recent advances in computational organoid models now allow simulation of complex tissues, including intestinal, lung, and pancreatic organoids (55, 98, 118). As *in vitro* organoid NAMs improve, integrating their insights (e.g., signaling-pathway dynamics, gene-expression feedback loops, cytokine release profiles, etc.) into computational organoid models will enhance the ability of these models to support early-phase therapeutic screening.

Like all modeling approaches, computational NAMs are only as robust as the data, assumptions, and mechanistic understanding that inform them. Furthermore, researcher-driven choices—such as parameter selection, as illustrated by early epidemiological models during the COVID-19 pandemic—can undermine model performance and lead to misleading interpretations (119, 120). The discovery of novel mechanistic insights necessitates researchers to revisit and update models with this new information, as exemplified by Montes-Olivas et al. in their work on intestinal organoid models. Advances in both the knowledge of the biochemical cues generating the organoids and the algorithms to detect them led to a more refined and insightful model of the system (121). The theoretical foundations of computational NAMs require continued experimental validation. Ongoing efforts should continue to focus on integrative models that combine genomic, structural, and phenotypic data to more accurately simulate complex virus-host dynamics.

### NAMs based on non-mammal systems

While human-based *in vitro* and computational NAMs are rapidly expanding, some biological questions still require whole-organism context. Non-mammalian vertebrates, such as nematodes (*Caenorhabditis elegans*) and zebrafish (*Danio rerio*), offer a valuable middle ground, providing robust, ethical, and scalable *in vivo* platforms that reduce reliance on traditional mammalian models (Fig. 1D). The importance of such approaches became particularly evident during the COVID-19 pandemic, when several nucleoside analog inhibitors targeting the SARS-CoV-2 RNA-dependent RNA polymerase (RdRp) showed therapeutic promise but raised concerns about fetal toxicity that precluded their use in pregnant patients (122–124). A recent study by Shiraki et al. leveraged the *C. elegans* system to screen for reproductive toxicity of RdRp inhibitors and reported strong concordance with adverse outcomes observed in animal models (125). This success reflects the nematode model’s conserved cellular and developmental pathways that are shared with mammalian systems. However, because *C. elegans* is not naturally susceptible to human viral pathogens, its utility in virology is largely limited to toxicity screening and genetic pathway analysis.

Zebrafish, by contrast, can support infection with multiple human pathogens and enable direct visualization of virus-host interactions in a whole-organism context. For example, zebrafish have been used to model infection with herpes simplex virus type 1 (126), Chikungunya virus (127), Zika virus (128), and human norovirus (129), among others (reviewed in [130]). Additionally, work by Gabor et al. demonstrated that the neuraminidase inhibitor Zanamivir retains anti-influenza A virus activity in zebrafish (131), underscoring the potential of this model for antiviral efficacy and screening studies. Despite these advantages, zebrafish are not universally compatible with human viral pathogens, as differences in optimal replication temperature, host range, and viral receptor conservation can restrict infectivity. Recognizing these limitations ensures that zebrafish are deployed where they provide the most biological insight while complementing—rather than replacing—other essential model systems.

## WHERE ANIMAL MODELS CONTINUE TO EXCEL

Despite advances in NAMs, animal models remain essential for studying complex physiological processes that cannot yet be fully captured *in vitro* or *in silico*. Key multicellular interactions—such as immune cell trafficking, endocrine and metabolic signaling, vascular responses, microbiome dynamics, and organ-to-organ communication—depend on whole-organism biology that current NAM platforms cannot reproduce (132, 133). These integrated processes critically shape host outcomes, particularly in the context of chronic or progressive diseases. For instance, for many oncogenic viruses, infection alone is not sufficient for carcinogenesis. A prime example is human papillomavirus (HPV), where nearly all sexually active adults will likely contract the virus at some point in their lifetime (134), yet only a small subset progress to cancer—a process that can take decades. Identifying the factors that contribute to malignant progression is, therefore, essential, and several studies point to the microbiome as playing a potential role (reviewed in [135]). Thus, animal models will be invaluable for dissecting the complex interplay between the virus, host, and microbiome (136, 137). For example, Spurgeon et al. leveraged the recently discovered murine papillomavirus, MmuPV1, to demonstrate that viral infection altered the microbiome of the female reproductive tract and impacted disease development (138). Such studies represent essential first steps in understanding disease progression and establishing robust models for preclinical therapeutic development (139).

Equally important, animal models allow investigators to account for host-level heterogeneity, including sex-dependent physiology (140–145), nutritional status (146–149), circadian rhythms, physical activity, and environmental exposures (150–153), all of which can shape viral pathogenesis and disease outcomes. For example, Chuong et al. used mouse models to demonstrate that nutritional status modulates disease severity following dengue virus infection (147). These dimensions of host biology remain difficult to capture in current NAM platforms, whereas animal models allow interrogation of questions that depend on integrated and dynamic host physiology. Further investigation of these important biological factors *in vivo* may yield important insights into viral disease mechanisms and identify previously unrecognized determinants of pathogenesis.

Animal models are uniquely suited for studying long-term viral infections and evaluating therapeutic strategies. For instance, retroviruses, such as HIV-1 and HTLV-1, as well as persistent herpesviruses like varicella-zoster virus (VZV), can remain quiescent for decades without overt symptoms. A major challenge in eradicating persistent infections lies in strategies aimed at suppressing latency and enhancing immune-mediated clearance. While *in vitro* cell-based models can interrogate some aspects of these processes, latently infected cells predominantly reside in specific tissues and anatomical compartments where drug penetration and immune accessibility vary. Consequently, the efficacy of latency-targeting interventions can only be fully evaluated using animal models. Bone marrow-liver-thymus (BLT) humanized mice represent one such model that has been effectively leveraged for evaluating strategies aimed at eradicating the latent reservoir of HIV-1 (154, 155).

Beyond viral latency, animal models are essential for understanding how infections impact long-term host physiology and contribute to post-acute sequelae. Diseases due to chronic infections often manifest with age-related changes in immunity, metabolism, and tissue repair, influencing disease progression and response to therapy. For example, VZV establishes lifelong latency in sensory neurons after primary infection, and reactivation due to age or other chronic stressors decades later can cause shingles, sometimes with neurological or systemic complications (156). By capturing these systemic and time-dependent effects, animal models provide a critical platform to study not only the natural history of infection but also the long-term safety and efficacy of antiviral or immunomodulatory interventions.

Finally, many aspects of antiviral and vaccine safety, efficacy, and pharmacokinetics can only be fully assessed in whole-animal systems. Determining how a drug is absorbed, metabolized, and cleared or how immune memory develops and persists is a challenge that cannot be fully predicted from NAMs alone. For example, the *in vivo* metabolism and dose-limiting pulmonary toxicity of remdesivir were identified in rhesus macaques during SARS-CoV-2 studies (157). Oseltamivir-resistant pandemic H1N1/2009 influenza virus (“swine flu”) transmissibility was studied in ferrets, where human-like respiratory physiology helps capture transmission-linked selective pressures (158). The use of whole organism systems can also reveal unanticipated off-target effects and tissue-specific responses. Adenovirus-vectored vaccines demonstrated rare, but clinically relevant, hepatic and thrombotic complications in mice and nonhuman primates, which were not predicted from cell culture or computational platforms (159, 160). Moreover, therapeutic outcomes often depend on dynamic processes that involve cytokine signaling, immune cell trafficking, vascular permeability, and organ-to-organ communication. For instance, HIV-1 humanized mouse models have demonstrated how lymphoid tissue architecture and microbiome-dependent immune activation help shape the viral reservoir and persistence (161). For these reasons, animal models remain the gold standard for validating the real-world performance of antiviral agents and vaccines and ensuring their safety and efficacy under physiologically authentic conditions.

## BALANCING NEW METHODS WITH ANIMAL MODELS

As human-relevant technologies advance, the role of animal models should evolve rather than disappear. The future of biomedical research will rely on integrated strategies that combine NAMs with targeted, judicious use of animals to answer questions that remain inaccessible to non-animal systems.

One emerging framework centers on three interconnected priorities. First, NAMs should be deployed in areas where they offer clear advantages. This includes pre-clinical development, high-throughput screening, and mechanistic evaluation under controlled environmental conditions (Table 1). Animal studies should then be reserved for questions that could not be addressed using NAMs alone. Second, continued progress toward reducing animal use requires strengthening NAMs themselves. Current protocols for NAM generation vary widely, complicating reproducibility and cross-study comparisons. By improving consistency in how NAMs are generated and providing community standards for NAM validation, researchers will enhance reproducibility and enable broader adoption of these new technologies. Third, incorporating NAMs into tiered research pipelines—beginning with *in silico* modeling, advancing to *in vitro* and/or *ex vivo* systems, and moving to animal studies when necessary for questions involving complex physiology—offers a practical roadmap for integrated research design.

**TABLE 1:** Comparison of NAM platforms for human virus research

Approach	Description	Advantage	Limitation	Examples
Primary cell cultures	Cell populations isolated directly from tissues; cultured *in vitro*	Preserve native cell behavior	Limited lifespan; reduced multicellular complexity	ALI cultures model epithelial structure and mucociliary action (15, 16, 31, 162)
Precision-cut tissue slices	Thin, intact sections of tissue prepared with specialized equipment	Slices maintain the native 3D architecture and cell types of complex tissue	Standardization is difficult; donor variability affects quality	PCLS used for drug screening and mechanistic studies (38, 40, 41)
Renewable cell cultures	iPSCs differentiate into germ layers and specialized cell types	Accessible, scalable, genetically tractable; strong for mechanistic studies	Time- and labor-intensive; potential genetic instability	Airway organoids support antiviral screening (52, 55, 163, 164)
Organ-on-chip	Microfluidic culture of engineered or native tissues	Captures flow/perfusion and mechanical forces	Technically complex; limited systemic physiology	Airway-on-a-chip models simulate virus infection and therapeutic testing (57, 58)
Computational biology	Data-driven predictive modeling	Rapid screening and hypothesis generation	Data and assumption-driven; bias-prone	Outbreak forecasting; vaccine design support (109, 112, 114, 115)
Non-mammal species	Lower ethical burden *in vivo* vertebrate models	Scalable *in vivo* screening; low cost; simple husbandry	Limited translational relevance and permissiveness to animal viruses	Zebrafish for IAV infection and antiviral testing (131)

Within this hybrid research paradigm, NAMs and animal models function not as competing alternatives, but as complementary pieces of a unified experimental strategy. Importantly, implementing such integrated frameworks will require coordinated efforts across laboratories, leveraging diverse technical expertise and collaborative study design. When adopted collectively, these approaches provide a robust, ethical, and translationally relevant foundation for advancing virology research, enabling scientific progress that neither NAMs nor animal models could achieve alone.
